# Impact of clinical and sociodemographic factors on fatigue among patients with substance use disorder: a cohort study from Norway for the period 2016–2020

**DOI:** 10.1186/s13011-020-00334-x

**Published:** 2020-12-14

**Authors:** Jørn Henrik Vold, Rolf Gjestad, Christer F. Aas, Fatemeh Chalabianloo, Svetlana Skurtveit, Else-Marie Løberg, Kjell Arne Johansson, Lars Thore Fadnes, for the INTRO-HCV Study Group, Christer F. Aas, Vibeke Bråthen Buljovcic, Fatemeh Chalabianloo, Jan Tore Daltveit, Silvia Eiken Alpers, Lars T. Fadnes, Trude Fondenes Eriksen, Per Gundersen, Velinda Hille, Kristin Holmelid Håberg, Kjell Arne Johansson, Rafael Alexander Leiva, Siv-Elin Leirvåg Carlsen, Martine Lepsøy Bonnier, Lennart Lorås, Else-Marie Løberg, Mette Hegland Nordbotn, Cathrine Nygård, Maria Olsvold, Christian Ohldieck, Lillian Sivertsen, Hugo Torjussen, Jørn Henrik Vold, Jan-Magnus Økland, Tone Lise Eielsen, Nancy Laura Ortega Maldonado, Ewa Joanna Wilk, Ronny Bjørnestad, Ole Jørgen Lygren, Marianne Cook Pierron, Olav Dalgard, Håvard Midgard, Svetlana Skurtveit, Aaron G. Lim, Peter Vickerman

**Affiliations:** 1 Department of Addiction Medicine, Haukeland University Hospital, Jonas Lies vei 65, N-5021 Bergen, Norway; 2 Department of Global Public Health and Primary Care, University of Bergen, Bergen, Norway; 3 Department of Psychiatry, Haukeland University Hospital, Bergen, Norway; 4 Department of Mental Disorders, Norwegian Institute of Public Health, Oslo, Norway; 5 Norwegian Centre for Addiction Research, University of Oslo, Oslo, Norway; 6 Department of Clinical Psychology, University of Bergen, Bergen, Norway

**Keywords:** Substance-related disorders, Fatigue, Fatigue severity scale, Quality of life, Comorbidities, Illicit drugs, Viral human hepatitis, HIV, Kidney disease

## Abstract

**Background:**

The impact of clinical and sociodemographic factors on fatigue remains unknown among patients with substance use disorders (SUD). This study aims to evaluate fatigue among patients with SUD using a nine-item fatigue severity scale (FSS-9) and identify the impact that clinical and sociodemographic factors – such as injecting substance use, chronic infectious diseases, liver fibrosis, opioid agonist therapy (OAT), debt difficulties, and housing situation – have on fatigue.

**Methods:**

We used data from a cohort of patients with SUD in Norway with annual health assessments surveying FSS-9 and some clinical and sociodemographic factors. A total of 915 FSS-9 measurements were collected from 654 patients during the period 2016–2020. We defined baseline as the first annual health assessment when the health assessments were listed chronologically. Time was defined as years from baseline. We used a linear mixed model to analyse whether the clinical and sociodemographic factors affected the FSS-9 sum score, presented with beta coefficients (β) with 95% confidence intervals (CI).

**Results:**

The mean sum score of the FSS-9 was 43 (standard deviation: 16) at baseline. Females compared with males (adjusted mean difference of FSS-9 sum score: 4.1, 95% CI: 1.3–7.0), having debt difficulties compared with having no debt difficulties (2.9;0.4–5.3), and frequent use of benzodiazepines (5.7;3.0–8.4) or amphetamines (-5.0;-8.0– -2.0) compared to less frequent or no use of these substances changed the FSS-9 baseline sum score. The other clinical and sociodemographic factors did not predict any clinically relevant change in the FSS-9 sum score from baseline to the following health assessments.

**Conclusion:**

Patients with SUD suffer from high levels of fatigue. Female patients, patients with debt difficulties, and those with extensive use of benzodiazepines are at particular risk of being fatigued. This should be taken into consideration when planning health services.

**Supplementary Information:**

The online version contains supplementary material available at 10.1186/s13011-020-00334-x.

## Background

Patients with Substance Use Disorders (SUD) suffer from a broad range of health-related difficulties that may contribute to fatigue [1–3]. Fatigue presents itself as a persistent and overwhelming feeling of exhaustion and loss of energy. The condition is mainly associated with chronic diseases and may mitigate treatment adherence and exacerbate comorbid disorders [4, 5]. In SUD populations, a myriad of external factors can interact with fatigue and affect these patients’ general well-being [6–8]. Injecting substance use, internal organ dysfunctions (predominantly kidney and liver diseases), mental disorders, as well as low income, unemployment, and homelessness are some of the external factors that interact with fatigue. Despite this, relatively little attention has been paid to the extent of fatigue and how much various external factors influence fatigue among patients with SUD. Therefore, understanding the key factors affecting fatigue is essential to improve treatment outcomes and adherence in this population.

Fatigue is associated with several sociodemographic and clinical factors. Among patients with the Hepatitis C Virus (HCV) infection, 50–70% have reported fatigue [9–11], while 33–88% of those with the Human Immunodeficiency Virus (HIV) infection have presented the same symptom [12]. A more uncertain prevalence of fatigue is seen among patients with the Hepatitis B Virus (HBV) [13, 14]. In addition, females, patients with lower educational levels, and those with opioid use disorders undergoing Opioid Agonist Therapy (OAT) with methadone or buprenorphine generally have a greater risk of fatigue [15, 16]. Disentangling the effects of the potential factors influencing fatigue in patients with SUD is essential for individualised treatment and developing clinical guidelines.

Fatigue is a subjective concept, and various definitions and instruments are used in the literature to capture it, which makes interpretations more complicated [17–19]. The nine-item fatigue severity scale (FSS-9) is a well-known questionnaire used to quantify fatigue treatment effects. It shows excellent validity and reliability across various chronic neurological and infectious diseases, such as multiple sclerosis [17], HCV infection [20], stroke [5], and Parkinson’s disease [21]. The fact that FSS-9 shows a high consistency across various chronic diseases makes it particularly suitable to estimate fatigue among patients suffering from SUD with complex and challenging comorbidities.

Thus, this prospective cohort study aims to investigate fatigue using the nine-item Fatigue severity scale (FSS-9) among patients with substance use disorders (SUDs) and predict the impact of sociodemographic and clinical factors on FSS-9, including educational level, housing situation, debt difficulties, chronic infectious diseases, injecting substance use, substance use, liver fibrosis, and kidney disease. Moreover, we estimate:

1. using annual health assessments, the FSS-9 sum score and whether and to what extent the sociodemographic and clinical factors impact this score;
2. the impact of sociodemographic and clinical factors on changes in the FSS-9 sum score from the first health assessment to the following annual health assessments;
3. for two separate subgroups – patients receiving methadone as opioid agonist therapy (OAT) and those receiving buprenorphine as OAT – the FSS-9 sum score, whether and to what extent the sociodemographic and clinical factors affect the FSS-9 sum score at baseline, and any changes in the FSS-9 sum score from the first health assessment to the following annual health assessments.

## Methods

### Data source

We used data from a cohort nested to the INTRO-HCV trial on patients with SUD in Bergen and Stavanger, Norway [22]. We collected data from May 2016 to January 2020, and recruited patients on OAT from outpatient clinics in Bergen and Stavanger, as well as patients with various SUDs receiving primary healthcare from the municipality clinics in the city of Bergen.

### Data collections

All included patients were assessed yearly with a health assessment, including FSS-9 measurements, sociodemographic data, and current substance use. Additionally, blood samples and liver fibrosis measurements using transient elastography were conducted. We collected all data in a health register using electronic data collection software (Checkware®) under research nurses’ supervision. All the clinical data, including information regarding OAT, OAT medication, substance use, and possible comorbid clinical conditions, were collected from the electronic medical record.

### Study sample

We included 915 FSS-9 measurements from 654 patients in the study period. In total, 225 had follow-up data and conducted the health assessment, including the FSS-9 questionnaire, twice (*n* = 188) or thrice (*n* = 37), providing 487 repeated measurements. The median time interval between the baseline health assessments, and any subsequent assessments in the same patients, including FSS-9 measurements, was 11 months (interquartile range (IQR): 9–14) (Additional file 1).

### Measuring fatigue

We measured fatigue during the last week using FSS-9, including items considering: mental and physical functioning, motivation, carrying out duties, and interference with work, family, or social life. An FSS-9 measurement was completed when all nine items in the questionnaires were entirely conducted during an annual health assessment. The FSS-9 items were answered on a Likert scale – ranging from 1 (no fatigue) to 7 (worst fatigue) – that demonstrates the fatigue level. A high score of FSS-9 items notes a high level of fatigue, while a mean FSS-9 item score greater than 4.0 revealed severe fatigue. The data collection software only allowed valid responses to each question and prompted empty questions before submission to minimise missing data. The FSS-9 was also translated and back-translated from the US-English version into Norwegian by qualified native Norwegian-speaking translators (Additional file 2) [23].

### Measuring liver stiffness and assessing blood samples

We assessed liver stiffness using transient elastography (Fibroscan®) to reveal liver fibrosis and cirrhosis. The elastography was reported as a median score of 10 measurements conducted by research nurses. A liver stiffness above 10 kilopascals (kPa) was defined as liver fibrosis, while a value above 12.5 kPa indicated liver cirrhosis [24]. We also collected blood samples, including hemoglobin, thrombocytes, C - reactive protein, aspartate aminotransferase, estimated glomerular filtration rate, hepatitis B surface antigen, HIV antigen/antibodies, HCV antibodies, and HCV polymerase chain reaction (HCV PCR) during the annual health assessment. Liver stiffness was estimated by calculating the AST to platelet ratio index (APRI) score and using transient elastography (Fibroscan®) (Additional file 3). Moreover, the hematological and biochemical samples were analysed to detect anemia (Hemoglobin), infection or inflammation (C – reactive protein), kidney disease (estimated glomerular filtration rate), liver disease (APRI), or chronic infectious diseases (HIV, HCV, and HBV), which could affect the FSS-9 score. Both elastography and blood samples were examined annually and simultaneously when conducting the annual health assessments. We analysed the blood samples at the Department of Laboratory Medicine, Haukeland University Hospital, Bergen, Norway, and at the Department of Medical Biochemistry and Microbiology, Stavanger University Hospital, Stavanger, Norway (accredited by ISO-standard 15,189).

### Definition of study variables, including sociodemographic and clinical factors

We defined baseline for patients as the first annual health assessment that included an FSS-9 measurement when we listed the health assessments chronologically. We dealt with each FSS-9 measurement as a sum score by summarising the value (one to seven) from each item and as a mean score calculated by dividing the sum score by nine (nine items). We defined being on OAT according to whether patients received buprenorphine or methadone (OAT opioids) at baseline. Further, in accordance with the World Health Organization’s standards, we calculated the daily dose of received OAT opioids as a ratio between the received dose per day divided by the expected mean dose of OAT opioids (buprenorphine 18 mg, buprenorphine-naloxone 18/4.5 mg or methadone 90 mg) [25]. We categorised educational level into five groups: ‘not completed primary school,’ ‘completed primary school (nine years),’ ‘completed high school (12 years),’ ‘three or fewer years of college or university’ or ‘more than three years of college or university.’ Patients’ housing situations in the 30 days prior to the FSS-9 measurement were classified into two groups: “stable” and “unstable.” The latter category involved patients who had lived on the street, in a homeless shelter, or with family and friends during the past 30 days. Others who had a more permanent residence were classified as having a stable housing situation. Debt difficulties were defined as striving with paying off legal or illegal debt due to a constrained private economy. We set ‘injecting substance use’ as having injected at any time during the past 12 months, whereas frequent substance use was categorised as consuming at least one of the substance groups, including ‘benzodiazepines or z-hypnotics,’ ‘cannabis,’ ‘stimulants (amphetamines or cocaine),’ ‘alcohol,’ and ‘heroin or other illicit opioids’, more than weekly during the 12 months prior to a health assessment. Patients who did not use substances or used them less than weekly during the past 12 months were categorised as having ‘no frequent use of substance’. Having chronic infectious diseases was defined as detecting HCV PCR (HCV), hepatitis B surface antigen (HBV), or HIV antigen/antibodies (HIV) in the blood samples. For HCV PCR, we used the Helmert contrast in order to classify patients into two groups – transmitted and non-transmitted – and further into two subgroups: whether patients have a low viral load (< 800,000 IU/ml) or high viral load (≥ 800,000 IU/ml). By this two-fold division, we investigated whether the level of viral HCV load was associated with changes in the fatigue level.

### Statistical analyses

We used Stata/SE 16.0 (StataCorp, TX, USA) for descriptive analysis and IBM SPSS version 26.0 for expectation-maximisation imputation and linear mixed model analyses. The threshold for statistical significance was set to *P* < 0.05 for all analyses unless otherwise stated. In all analyses, we defined time as years from baseline.

We dealt with any missing values concerning sociodemographic and clinical factors – such as educational level, housing situation, debt difficulties, receiving OAT, OAT opioid dose ratio, injecting substance use, substance use, and the results of defined blood samples and transient elastography – as ‘missing at random’ when running expectation-maximisation imputation. We identified missing values in 2.6% in these factors and all were replaced with estimated values by imputation.

The FSS-9 sum score at baseline was calculated by summarising the nine items’ points. Linear mixed model analyses were used to investigate whether the sociodemographic and clinical factors affected the FSS-9 sum score and to what extent they impacted any changes in the score from baseline to following the health assessments. First, the factor variables were analysed separately as outcome variables as a function of the time (time from baseline). We did not identify substantial significant changes in the sociodemographic and clinical factors between the annual health assessments (data not shown). Thus, baseline levels were used as stable predictors in the prediction of the level and changes in FSS-9. We specified the linear mixed models as a random intercept fixed slope regression model. The estimator was set to Restricted Maximum Likelihood. To explore whether predictors predicted changes in outcome, the interactions between these factors and time were added to the model. The full information maximum likelihood ensured that all available FSS-9 sum score measurements were used. Additionally, we presented sub-group analyses for OAT patients using methadone or buprenorphine, respectively. For these analyses, we added the OAT opioid ratio as a predictor. The potential correlations between sociodemographic and clinical factors and fatigue are presented in Additional file 4. We performed a sensitivity analysis by adding Bonferroni corrected *p*-values to adjust for Type I errors in all analyses.

### Ethics approval and consent to participate

The study is reviewed and approved by the Regional Ethical Committee for Health Research West, Norway (REK Vest 2017/51). Each patient provided written informed consent prior to enrolling in the study.

## Results

### Patients characteristics at baseline

Seventy-one percent of patients were male, and the mean age was 43 years (standard deviation (SD): 11 years) at baseline (Table 1). Six percent had not completed primary school, or 44% had primary school as their highest educational level. 82% received OAT, of which 60% received buprenorphine or buprenorphine-naloxone as an OAT opioid. Further, 13% had an unstable housing situation in the last 30 days leading up to the FSS-9 measurement. 73% had used at least one substance weekly during the past 12 months.

**Table 1:** Sociodemographic and clinical characteristics at baseline for all patients and for patients with more than one annual health assessment

	All patients (***N*** = 654)	Patients with ≥2 health assessments (***N*** = 225)
*Age (years), n (%)*		
18–29	81 (12)	23 (10)
30–39	185 (28)	63 (28)
40–49	205 (31)	75 (33)
50–59	148 (23)	53 (24)
≥ 60	35 (5)	11 (5)
Mean (SD)	43 (11)	44 (10)
Gender, n (%)		
Male	461 (71)	170 (76)
Female	193 (29)	55 (24)
Highest educational level, n (%)		
Not completed primary school	40 (6)	15 (7)
Completed primary school (9 years)	286 (44)	105 (47)
Completed high school (12 years)	259 (40)	81 (36)
≤ 3 years of college or university	57 (9)	20 (9)
> 3 years of college or university	12 (2)	<5 (2)
*Receiving opioid agonist therapy, n (%)*	537 (82)	205 (91)
*OAT opioid (%)*		
Methadone	209 (39)	96 (43)
Buprenorphine/Buprenorphine-naloxone	321 (60)	107 (48)
*OAT opioid dose ratio (median (IQR))*^a^	0.9 (0.8–1.1)	1.0 (0.9–1.1)
*Housing situation the past 30 days, n (%)*		
Stable^b^	569 (87)	203 (90)
Unstable^c^	85 (13)	22 (10)
*Injected substances the past 12 months, n (%)*	338 (56)	116 (52)
*Frequent substance use the past 12 months, n (%)*^d^		
Alcohol	154 (26)	56 (25)
Benzodiazepines	238 (39)	87 (39)
Cannabis	313 (52)	124 (55)
Opioids	97 (16)	27 (12)
Stimulants (amphetamines and cocaine)	176 (29)	60 (27)
*Chronic infectious diseases, n (%)*		
Hepatitis C virus infection	315 (48)	184 (82)
Low virulent (< 800,000 IE/ml)	168 (25)	92 (41)
High virulent (≥ 800,000 IE/ml)	147 (22)	92 (41)
Hepatitis B virus infection	5 (0)	< 5 (< 1)
Human immunodeficiency virus	< 5 (< 1)	< 5 (< 1)
*Hematological and biochemical samples, median (IQR)*		
Hemoglobin (g/dl)	14 (13–15)	14 (13–15)
Estimated glomerulus filtration rate (ml/min/1.73 m^2^)	104 (89–122)	105 (91–124)
C-reactive protein (mg/L)	4 (1–9)	3 (1–8)
Aspartate transaminase (U/L)	31 (23–50)	40 (30–65)
*Liver stiffness, median (IQR)*		
Transient elastography (kPa)	5 (4–7)	6 (5–8)
Aspartate transaminase to platelets ratio index	0.3 (0.2–0.6)	0.4 (0.3–0.8)

The table displays the sociodemographic and clinical characteristics for all included patients, and for patients with two or more health assessments, including FSS-9 measurements at baseline

*FSS-9* nine-item fatigue severity scale (Likert scale), *IQR* interquartile range, *kPa *kilopascal, *OAT* opioid agonist therapy, *SD* standard deviation

^a^OAT opioid ratio is a ratio between the received dose of OAT opioids per day and the expected median daily dose (18 mg buprenorphine, 18/4.5 mg buprenorphine-naloxone or 90 mg methadone). A ratio on 1.0 indicates that patients received the expected daily dose; ^b^A stable housing situation was defined as having owned or rented housing situation or being imprisoned; ^c^Unstable housing situation was defined as living in a homeless shelter, with family or friends, or on the street; ^d^Frequent substance use was defined as using substance at least weekly during the past 12 months

#### FSS-9 sum scores at baseline

The mean sum score for the FSS-9 was 43 (SD: 16), representing a mean score for the FSS-9 items of 4.8 (2.6) (Table 2). A total of 69% of patients had severe fatigue. The mean FSS-9 sum score was slightly left-skewed (skewness: − 0.7) and tended towards a flattened distribution (kurtosis: 2.4).

**Table 2:** Mean (Standard deviation (SD)) item scores for single items on FSS-9 at baseline and follow-up

	***Baseline (N = 654)***	***Follow-up (N = 225)***
**FSS-9**		
I1: My motivation is lower when I am fatigued	5.4 (2.0)	5.6 (2.0)
I2: Exercise brings on my fatigue	4.7 (2.1)	5.0 (2.0)
I3: I am easily fatigued	4.5 (2.1)	4.8 (2.1)
I4: Fatigue interferes with my physical functioning	4.9 (2.1)	5.1 (2.0)
I5: Fatigue causes frequent problems for me	4.4 (2.2)	4.5 (2.2)
I6: My fatigue prevents sustained physical functioning	4.6 (2.2)	4.4 (2.2)
I7: Fatigue interferes with carrying out certain duties and responsibilities	5.0 (2.1)	5.0 (2.1)
I8: Fatigue is among my three most disabling symptoms	4.6 (2.3)	4.8 (2.3)
I9: Fatigue interferes with my work, family, or social life	4.9 (2.2)	4.6 (2.3)
Mean score of all items	4.8 (1.8)	4.9 (1.7)
Sum score of all items	43.2 (15.9)	43.8 (15.2)

Follow-up: FSS-9 score on the last health assessment during the study period among patients with two or more annual health assessments; *FSS-9* nine-item fatigue severity scale (Likert scale ranging from 1 (no fatigue) to 7 (worst fatigue)), *I* Item, *SD* standard deviation

The mean scores for the FSS-9 were 43 (SD: 16) for patients receiving methadone and 43 (17) for those using buprenorphine (Additional file 5), corresponding to a mean score for the FSS-9 items of 4.8 (1.8) for patients receiving methadone and 4.7 (1.9) for those using buprenorphine. Severe fatigue was identified in 77% of patients receiving methadone and 67% of those using buprenorphine. In these two sub-groups, the FSS-9 sum scores were slightly left-skewed (skewness: − 1.0 (methadone group), − 0.6 (buprenorphine group)) and flattened distributed (kurtosis: 3.0 (methadone group), 2.2 (buprenorphine group)).

#### The sociodemographic and clinical factors’ impact on the FSS-9 sum score at baseline and the factors’ influence on changes in the FSS-9 sum score from baseline to the following annual health assessments

At baseline, we found that the FSS-9 sum score was higher for females than males (adjusted mean FSS-9 sum score difference: 4.1, CI 1.3;7.0, *p* = 0.005), for patients with debt difficulties compared with those without debt difficulties (2.9, CI 0.4;5.3, *p* = 0.022), and for patients with frequent benzodiazepine use compared with those with less frequent or no use (5.7, CI 3.0;8.4, *p* < 0.001) (Table 3). In contrast, the FSS-9 sum score was lower for patients with frequent stimulant use than those with less frequent or no use (− 5.0, CI -8.0;-2.0, *p* = 0.001). Moreover, we saw a small non-clinical significant reduction of the FSS-9 sum score from baseline to the following annual health assessments for patients with frequent benzodiazepine use compared to those with less frequent or no use (− 4.4, CI -8.2;-0.7, *p* = 0.021) and for patients having significant liver fibrosis or cirrhosis measured by transient elastography compared with those with non-significant fibrosis or normal liver stiffness (− 5.5, CI -9.9;-1.0, *p* = 0.016). With Bonferroni corrected *p*-values, we only found that patients with frequent benzodiazepine or stimulant use compared with those with less frequent or no use of these substances changed the fatigue levels at baseline.

**Table 3:** Linear mixed model of fatigue (FSS-9) adjusted for sociodemographic and clinical factors (N = 654)

Fixed effects				
	Effect estimate		Time trend (per year)	
	Estimate (95% CI)	*p*-value	Slope (95% CI)	*p*-value
FSS-9 sum score	42 (26–58)	< .001	3.6 (−23.5–30.7)	0.792
Female	*4.1 (1.3–7.0)*	*0.005*	−0.3 (−4.5–3.8)	0.877
Age per 10 years^1)^	0.2 (−1.0–1.4)	0.755	−0.2 (− 2.1–1.7)	0.844
Educational level	− 1.1 (− 2.6–0.3)	0.132	−0.3 (− 2.3–1.7)	0.754
Unstable housing situation	0.0 (−3.7–3.7)	0.992	2.6 (− 3.6–8.8)	0.408
Debt difficulties	*2.9 (0.4–5.3)*	0.022	−0.2 (− 3.8–3.3)	0.898
Injecting substance use	−0.1 (− 2.9–2.7)	0.944	−0.7 (− 4.6–3.3)	0.740
*Frequent use of substances*				
Benzodiazepines	*5.7 (3.0–8.4)*	*< .001*^*a*^	*−4.4 (−8.2* – − 0.7)	*0.021*
Alcohol	1.8 (−1.1–4.6)	0.221	0.6 (−3.5–4.7)	0.776
Cannabis	1.2 (− 1.4–3.8)	0.356	1.8 (− 1.7–5.3)	0.309
Opioids	3.3 (− 0.3–6.9)	0.069	−4.6 (− 10.8–1.7)	0.149
Stimulants^2)^	*− 5.0 (− 8.0*– *− 2.0)*	*0.001* ^*a*^	2.1 (− 2.1–6.3)	0.327
*Chronic infectious diseases*				
Hepatitis B virus infection	3.3 (− 10.4–16.9)	0.638	−2.6 (− 16.8–11.5)	0.715
Hepatitis C virus infection				
- Detected	3.0 (− 5.4–11.4)	0.484	0.7 (− 18.7–20.1)	0.941
- Low vs. high viral load	− 0.4 (− 10.3–10.9)	0.948	− 7.0 (− 17.1–3.0)	0.169
HIV	− 0.1 (− 15.3–15.5)	0.994	13.0 (− 6.8–32.7)	0.197
*Liver stiffness*				
Transient elastography per 10 kPa	1.2 (−1.6–4.0)	0.391	*−5.5 (− 9.9 – − 1.0)*	*0.016*
APRI score per 1 unit	0.5 (− 0.6–1.5)	0.378	1.4 (− 0.9–3.6)	0.230
*Hematologic and biochemical blood samples (continuous variables)*				
Hemoglobin per 1 unit (g/dL)	−0.3 (− 1.1–0.6)	0.513	0.3 (− 0.9–1.5)	0.622
eGFR per 30 units (ml/min/1.73m^2^)	0.0 (− 2.0–0.9)	0.453	0.0 (− 2.0–1.9)	0.973
CRP per 10 units (ml/L)	−0.1 (− 0.6–0.7)	0.848	0.0 (− 0.1–0.2)	0.682

The table displays a linear mixed model analysis (Restricted Maximum Likelihood regression) evaluating sociodemographic and clinical factors’ (predictors) changes in the FSS-9 sum score at baseline and the predictors’ influence on changes in the FSS-9 sum score (time trend) per year from baseline. The predictors’ effect estimates and time trends estimate adjusted mean differences in the FSS-9 sum score

*APRI* aspartate transaminase to platelet ratio index, *CI* confidence interval, *CRP* C-reactive protein, *FSS-9* nine-item fatigue severity scale, *eGFR* estimated glomerular filtration rate, *HIV* human immunodeficiency virus, *kPa* Kilopascal, *OAT* opioid agonist therapy

^1)^ Age per 10 years was centred according to mean age (43 years) in the study sample at baseline. ^2)^ Includes amphetamine or cocaine use. The educational level: highest level of education was coded 0–4 with 4 as the highest educational level. Unstable housing situation: living on the street, homeless shelter, or with family and friends at any time during the past 30 days prior to the health assessment. Debt difficulties: struggling with repaying current illegal and legal debt. Injecting substance use: Having injected substance during the past 12 months prior to the health assessment. Frequent use of substances: at least weekly during the past 12 months prior to the health assessment. Viral load of HCV: From − 0.5 to 0.5, where the range ≥ − 0.5 to < 0 represents the low viral load (HCV PCR < 800,000 IE/ml), and the range ≤ 0.5 to > 0 identifies the high viral load (HCV PCR ≥ 800,000 IE/ml). Zero (0) defined patients without HCV infection

^a)^ Statistically significant results when using Bonferroni corrected *p*-values (α_altered_ = 0.05 / 41 = 0.0012)

#### The sociodemographic and clinical factors’ impact on changes in the FSS-9 sum score from baseline to the following annual health assessments among patients on OAT

Among patients receiving methadone as an OAT opioid, we found that the FSS-9 sum score was higher for females than males (7.3, CI 2.5;12.2, *p* = 0.003), for patients having debt difficulties compared with those not having debt difficulties (4.9, CI 0.7;9.1, *p* = 0.023), for patients having frequent benzodiazepine use compared with those having less frequent or no use (6.0, CI 1.6;10.5, *p* = 0.008), and for patients with a high HCV viral load compared with those with a low HCV viral load (31.5, CI 1.5;61.5, *p* = 0.040) at baseline (Additional file 6). Among patients receiving buprenorphine as an OAT opioid, we found that patients with frequent alcohol use had higher the FSS-9 sum score (4.8, CI 0.2;9.3, *p* = 0.039), while patients with frequent stimulant use had lower the FSS-9 score (− 5.0, CI -9.9;-0.1, *p* = 0.047) compared with patients with less frequent or no use of these substances at baseline (Additional file 7). For both subgroups, no sociodemographic and clinical factors were clinically associated with substantial changes in the FSS-9 sum score from baseline to the following annual health assessments. With Bonferroni corrected *p*-values, we did not identify any predictors that changed the fatigue score at baseline and between the annual health assessments.

## Discussion

This study showed that 69% of SUD patients had severe fatigue symptoms. The sociodemographic and clinical factors that substantially contributed to higher fatigue scores at baseline were females compared with males (four points), frequent benzodiazepine use compared with less frequent or no use (six points), and debt difficulties compared with no debt difficulties (three points). However, the fatigue score was five points lower for patients with frequent stimulant use than those with less frequent or no use. For patients using buprenorphine as an OAT opioid, we found five points lower fatigue score for patients with frequent stimulant use and five points higher fatigue score for patients with frequent alcohol use compared with those with less frequent or no use of these substances at baseline. For patients receiving methadone as an OAT opioid, the fatigue score was higher for females than males (seven points), for patients with frequent benzodiazepine use compared with those with less frequent or no use (six points), for patients with debt difficulties compared with those without difficulties with debt (five points), and for patients with a high versus a low viral load of HCV (32 points) at baseline. The latter finding suggesting an extreme difference between a high and a low viral load of HCV was surprising. Other studies assessing HCV viral load and correlation based on clinical and histological features have also not found HCV viral load to impact other related outcomes [26–28]. This finding is likely related to random variability within the data, and it should be interpreted with caution. Moreover, no sociodemographic and clinical factors were associated with clinically significant changes in the fatigue score from baseline to the following health assessments.

In the present study, patients with SUD had a mean fatigue score (4.8) comparable to some of the most severe chronic diseases. In recent studies, patients infected with HIV or HCV, or those co-infected with both of them, have a mean FSS-9 score that ranged from 3.3 to 4.5 [10, 29, 30]. Patients with myasthenia gravis have reported a comparable fatigue score of 4.7 [31], and similarly, so have patients who have suffered from a stroke at least 6 months after the stroke onset (4.8) [32]. One can assume that a high prevalence of underlying mental disorders, extensive polysubstance use, and lower social status could have attributed to the high level of fatigue in the SUD population.

We found that females were weakly more fatigued than males. For both genders, patients with frequent use of benzodiazepines were weakly more fatigued than those with less frequent or no use. Recent studies evaluating fatigue in the general population and patients with chronic disorders have demonstrated similar gender differences in fatigue levels [15, 23, 31, 33]. Gender inequalities regarding household responsibilities and caring for the family have generally been highlighted as explanations for females’ fatigue levels [34]. Additionally, females with SUD may be worse off than males in many domains. They may have less financial resources, experience more physical trauma caused by exchanging sex for drugs and money, and face more stigma related to family failures [34]. Moreover, in the general population and among patients with SUDs, females are more likely to use benzodiazepines than males, with a similar tendency found in different countries [2, 35–38]. Females’ higher prevalence of anxiety disorders, sleeping disorders and the fact that they are more likely to seek medical care may contribute to more prescriptions of hypnotics and anxiolytics, such as benzodiazepines and z-hypnotics [39–41]. One can believe that these medical, psychological, and social challenges may overall explain the gender gap concerning a higher fatigue level among females in the SUD population.

Our findings revealed that patients with frequent use of benzodiazepines were weakly more fatigued than those with less frequent or no use. Among patients with frequent use of stimulants, we found that they were less fatigued than those with less frequent or no use. These group differences were overall small. In the general population, previous studies assessing the effect of benzodiazepine use have suggested that patients using benzodiazepines have a lower quality of life, self-reported physical health, and more disability than those not using benzodiazepines [42, 43]. This is parallel with the higher fatigue levels shown in the present study. Furthermore, using stimulants, particularly illicit amphetamines, is generally associated with poor mental health and stimulant withdrawal symptoms in the SUD populations [44, 45]. A temporary sense of better self-perceived mental health and fewer withdrawal symptoms may arise when consuming stimulant substances, which contributes to a temporary reduction of fatigue compared to the experience without stimulants. However, our trend analyses indicate that the fatigue levels remained stable over time when the frequent use of stimulants is persistent.

The present study showed no clear associations between fatigue and chronic infectious diseases or kidney disease. For patients with HBV, HIV or end-stage kidney disease, the low prevalence of HBV and HIV and a mean renal function within the normal range could explain why no associations with fatigue were detected. Furthermore, we are surprised that SUD patients with HCV infections did not demonstrate a higher fatigue level compared to patients with SUD without HCV infection, considering that the prevalence of fatigue is up to 70% in populations with HCV [9–11], which is considerably higher than in general population [23]. However, the large extent of polysubstance use in the present population (75%) could have temporarily displaced the HCV infection’s change on fatigue.

We found that 77% of patients using methadone as an OAT opioid and 67% of those using buprenorphine had severe fatigue symptoms. Four sociodemographic and clinical factors significantly changed fatigue levels among methadone users, while two factors influenced fatigue among those using buprenorphine. Methadone is a full opioid agonist associated with more euphoria and analgesia than the partial opioid agonist buprenorphine [46]. In a quantitative study evaluating patients’ experience of using methadone and buprenorphine in OAT, unwanted physical effects, for example, over-sedation, were particularly pointed out in some methadone cases [47]. These effects might explain methadone users’ high prevalence of severe fatigue symptoms and why more sociodemographic and clinical factors influenced methadone users’ the fatigue levels than those using buprenorphine.

Overall, no single sociodemographic and clinical factor was associated with substantial changes in fatigue at baseline or over time in the SUD population. This signifies that fatigue was substantially constant between patients. However, the mean fatigue level significantly exceeded the threshold for severe fatigue in the SUD population, which underlies the importance of identifying patients who simultaneously have several sociodemographic and clinical risk factors for severe fatigue. Identifying these patients and treating the underlying causes of fatigue should be the way to reduce the fatigue in the population.

## Strengths and limitations

This study has several strengths. We have included 654 patients with SUD that usually are difficult to reach in health care. Of those, 225 patients were followed up by two or three annual health assessments, making longitudinal analyses possible. This study does, however, have some limitations. First, the patients were mainly recruited from outpatient clinics receiving OAT. The majority had opioid dependence, although this was often combined with other dependencies, which could affect the generalisability of our results to other SUD populations. Second, we had a prospective follow-up of only a third of those patients recruited at baseline. This also causes weakness in our results and makes it necessary to carefully interpret the longitudinal analyses. Third, due to clinical challenges, including systematic and patient delays, the annual health assessments were not precisely conducted one year after the previous health assessment. This may affect the interpretation of the predicted fatigue level changes from baseline. Fourth, due to data imputation and inclusion of up to 42 predictors in the linear mixed model analyses, there is a risk of Type I error. We dealt with this by presenting the Bonferroni corrections to all *p*-values in the analyses.

## Conclusion

The present study shows a high symptom burden of fatigue among patients with SUDs, particularly among females, patients with debt difficulties, and those with extensive use of benzodiazepines. Identifying severe fatigue and considering fatigue in the follow-ups could help optimise SUD treatment for these patients. Policymakers could take this into consideration when planning health services.

## Supplementary Information

**Additional file 1.** The number of months from baseline to the second or third health assessment. No.: Number of patients; SD: Standard deviation; ref.: Reference. The table displays the number of patients with one, two, and three health assessments, including a nine-item Fatigue Severity Scale score. The table displays the time between baseline and the second and third health assessments.

**Additional file 2.** The US-English and the Norwegian versions of FSS-9. Description: Legends: FSS-9; Nine-item Fatigue Severity Scale. All items in the FSS-9 are ranged as a Likert scale from 1 to 7, where 1 indicates “strongly disagree” and 7 “strongly agree”.

**Additional file 3.** Aspartate aminotransferase to platelet ratio index (APRI). Description: APRI: Aspartate aminotransferase to platelet ratio index. The figure displays the APRI score equation. The AST upper limit of normal range was defined as 45 IU/L (male) and 35 IU/L (female).

**Additional file 4.** Potential correlations between sociodemographic and clinical factors and fatigue. The figure shows that potential sociodemographic and clinical comorbidities may affect fatigue among patients with substance use disorders.

**Additional file 5.** Mean (SD) item scores for single items on the FSS-9 at baseline and follow-up. Legend**:** FSS-9: Nine-item Fatigue Severity Scale; I: Item; OAT: Opioid agonist therapy; SD: Standard Deviation. Follow-up: The FSS-9 score on the last health assessment during the study period among patients with two or more annual health assessments. Mean (SD) item scores for single items on the FSS-9 for patients using methadone or buprenorphine as OAT opioid.

**Additional file 6.** Linear mixed model of fatigue (FSS-9) adjusted for sociodemographic and clinical factors among patients receiving methadone as an OAT opioid at baseline (*N* = 209). Legends**:** APRI: Aspartate transaminase to platelet ratio index; CI: Confidence interval; CRP: C-reactive protein; FSS-9: Nine-item Fatigue Severity Scale; eGFR: estimated glomerular filtration rate; HIV: Human Immunodeficiency virus; kPa: Kilopascal; OAT: Opioid Agonist Therapy. ^1)^ Age per 10 years was centred according to mean age (43 years) in the study sample at baseline. ^2)^ Includes amphetamine or cocaine use. The OAT opioid ratio: a ratio between the received dose of OAT opioids per day divided by the expected mean dose of OAT opioids (buprenorphine 18 mg, buprenorphine-naloxone 18/4.5 mg or methadone 90 mg). The educational level: highest level of education was coded 0–4 with 4 as the highest educational level. Unstable housing situation: living on the street, homeless shelter, or with family and friends at any time in the past 30 days prior to the health assessment. Debt difficulties: struggling with repaying current illegal and legal debt. Injecting substance use: Having injected a substance in the past 12 months prior to the health assessment. Frequent use of substances: at least weekly during the past 12 months prior to the health assessment. Viral load of HCV: From − 0.5 to 0.5, where the range ≥ − 0.5 to < 0 represents the low viral load (HCV PCR <  800,000 IE/ml), and the range from ≤0.5 to > 0 identifies the high viral load (HCV PCR ≥ 800,000 IE/ml). Zero (0) defined patients without HCV infection. When using the Bonferroni corrected *p*-values (α_altered_ = 0.05 / 43 = 0.0012), the predictors did not affect the FSS-9 sum score significantly. The table displays a linear mixed model analysis (Restricted Maximum Likelihood regression) evaluating sociodemographic and clinical factors’ (predictors) changes in the FSS-9 sum score at baseline and their influence on changes in the FSS-9 sum score (time trend) per year from baseline among patients receiving methadone as an OAT opioid. The predictors’ effect estimates and time trends estimate adjusted mean differences in the FSS-9 sum score.

**Additional file 7.** Linear mixed model of fatigue (FSS-9) adjusted for sociodemographic and clinical factors among patients receiving buprenorphine as an OAT opioid at baseline (*N* = 321). Legends**:** APRI: Aspartate transaminase to platelet ratio index; CI: Confidence interval; CRP: C-reactive protein; FSS-9: Nine-item Fatigue Severity Scale; eGFR: estimated glomerular filtration rate; HIV: Human Immunodeficiency virus; kPa: Kilopascal; OAT: Opioid Agonist Therapy. ^1)^ Age per 10 years was centred according to mean age (43 years) in the study sample at baseline. ^2)^ Includes amphetamine or cocaine use. The OAT opioid ratio: a ratio between the received dose of OAT opioids per day divided by the expected mean dose of OAT opioids (buprenorphine 18 mg, buprenorphine-naloxone 18/4.5 mg or methadone 90 mg). The educational level: highest level of education was coded 0–4 with 4 as the highest educational level. Unstable housing situation: living on the street, homeless shelter, or with family and friends at any time during the past 30 days prior to the health assessment. Debt difficulties: struggling with repaying current illegal and legal debt. Injecting substance use: Having injected a substance during the past 12 months prior to the health assessment. Frequent use of substances: at least weekly during the past 12 months prior to the health assessment. Viral load of HCV: From − 0.5 to 0.5, where the range ≥ − 0.5 to < 0 represents the low viral load (HCV PCR <  800,000 IE/ml), and the range ≤ 0.5 to > 0 identifies the high viral load (HCV PCR ≥ 800,000 IE/ml). Zero (0) defined patients without HCV infection. When using the Bonferroni corrected p-values (α_altered_ = 0.05 / 43 = 0.0012), the predictors did not affect the FSS-9 sum score significantly. The table displays a linear mixed model analysis (Restricted Maximum Likelihood regression) evaluating sociodemographic and clinical factors’ (predictors) changes in the FSS-9 sum score at baseline and their influence on changes in the FSS-9 sum score (time trend) per year from baseline among patients receiving buprenorphine as an OAT opioid. The predictors’ effect estimates and time trends estimate adjusted mean differences in the FSS-9 sum score.

## Data Availability

No additional data are available due to data protection requirements.

## Abbreviations

APRI: Aspartate transaminase to platelet ratio index
CI: Confidence interval
FSS-9: Nine-item Fatigue Severity Scale
HBV: Hepatitis B Virus
HCV: Hepatitis C Virus
HCV PCR: HCV Polymerase Chain Reaction
HIV: Human Immunodeficiency Virus
IQR: Interquartile Range
kPa: Kilopascal
OAT: Opioid Agonist Therapy
SUD: Substance Use Disorder
SD: Standard Deviation
