# Supplementary material for: Impact of clinical and sociodemographic factors on fatigue among patients with substance use disorder: a cohort study from Norway for the period 2016–2020
Source: Subst Abuse Treat Prev Policy. 2020 Dec 14;15:93. doi: 10.1186/s13011-020-00334-x (PMC7737389; doi:10.1186/s13011-020-00334-x)
Supplement: Supplementary file 1 — Additional file 1. The number of months from baseline to the second or third health assessment. No.: Number of patients; SD: Standard deviation; ref.: Reference. The table displays the number of patients with one, two, and three health assessments, including a nine-item Fatigue Severity Scale score. The table displays the time between baseline and the second and third health assessments. [file 13011_2020_334_MOESM1_ESM.docx]

**Additional File 1**

Title: The number of months from baseline to the second or third health assessment

| Health assessment | No. | Months after the baseline date | | | | |  |  |
| --- | --- | --- | --- | --- | --- | --- | --- | --- |
|  |  |  | Percentiles | | | | |  |
|  |  | Mean (SD) | | 25 | 50 | 75 | | |
| 1 (baseline) | 654 | 0 (ref.) | | 0 (ref.) | 0 (ref.) | 0 (ref.) | | |
| 2 | 225 | 11.6 (4.2) | | 8.7 | 10.9 | 14.0 | | |
| 3 | 37 | 18.6 (5.6) | | 15.6 | 18.3 | 24.3 | | |
