# Supplementary material for: Impact of clinical and sociodemographic factors on fatigue among patients with substance use disorder: a cohort study from Norway for the period 2016–2020
Source: Subst Abuse Treat Prev Policy. 2020 Dec 14;15:93. doi: 10.1186/s13011-020-00334-x (PMC7737389; doi:10.1186/s13011-020-00334-x)
Supplement: Supplementary file 2 — Additional file 2. The US-English and the Norwegian versions of FSS-9. Description: Legends: FSS-9; Nine-item Fatigue Severity Scale. All items in the FSS-9 are ranged as a Likert scale from 1 to 7, where 1 indicates “strongly disagree” and 7 “strongly agree”. [file 13011_2020_334_MOESM2_ESM.docx]

**Additional File 2**

Title: The US-English and the Norwegian versions of FSS-9

| I1: My motivation is lower when I am fatigued |
| --- |
| I2: Exercise brings on my fatigue |
| I3: I am easily fatigued |
| I4: Fatigue interferes with my physical functioning |
| I5: Fatigue causes frequent problems for me |
| I6: My fatigue prevents sustained physical functioning |
| I7: Fatigue interferes with carrying out certain duties and responsibilities |
| I8: Fatigue is among my three most disabling symptoms |
| I9: Fatigue interferes with my work, family, or social life |

| I1: Mitt pågangsmot blir dårligere når jeg er utmattet |
| --- |
| I2: Jeg blir fort utmattet ved anstrengelser |
| I3: Jeg har lett for å bli utmattet |
| I4: Utmattelse nedsetter min fysiske funksjonsevne |
| I5: Utmattelse skaper ofte problemer for meg |
| I6: Utmattelse fører til at jeg har dårlig fysisk utholdenhet over lengre tid |
| I7: Utmattelse virker negativt inn på mine gjøremål og forpliktelser |
| I8: Utmattelse er ett av mine tre mest plagsomme symptomer |
| I9: Utmattelse virker negativt inn på mitt arbeid, min familie og mitt øvrige sosiale liv |
