# Supplementary material for: Impact of clinical and sociodemographic factors on fatigue among patients with substance use disorder: a cohort study from Norway for the period 2016–2020
Source: Subst Abuse Treat Prev Policy. 2020 Dec 14;15:93. doi: 10.1186/s13011-020-00334-x (PMC7737389; doi:10.1186/s13011-020-00334-x)
Supplement: Supplementary file 3 — Additional file 3. Aspartate aminotransferase to platelet ratio index (APRI). Description: APRI: Aspartate aminotransferase to platelet ratio index. The figure displays the APRI score equation. The AST upper limit of normal range was defined as 45 IU/L (male) and 35 IU/L (female). [file 13011_2020_334_MOESM3_ESM.docx]

**Additional File 3**

*Title: Aspartate aminotransferase to platelet ratio index (APRI)*

$$APRI=\frac{\frac{AST level (\frac{IU}{L})}{AST \left( Upper limit of Normal \right) (\frac{IU}{L})}}{Platelet Count (\frac{{10}^{9}}{L})}x 100$$

AST Upper limit of Normal was defined as 45 IU/L (male) and 35 IU/L (female).
