# Supplementary figures and images for: Impact of clinical and sociodemographic factors on fatigue among patients with substance use disorder: a cohort study from Norway for the period 2016–2020
Source: Subst Abuse Treat Prev Policy. 2020 Dec 14;15:93. doi: 10.1186/s13011-020-00334-x (PMC7737389; doi:10.1186/s13011-020-00334-x)

Additional File 4

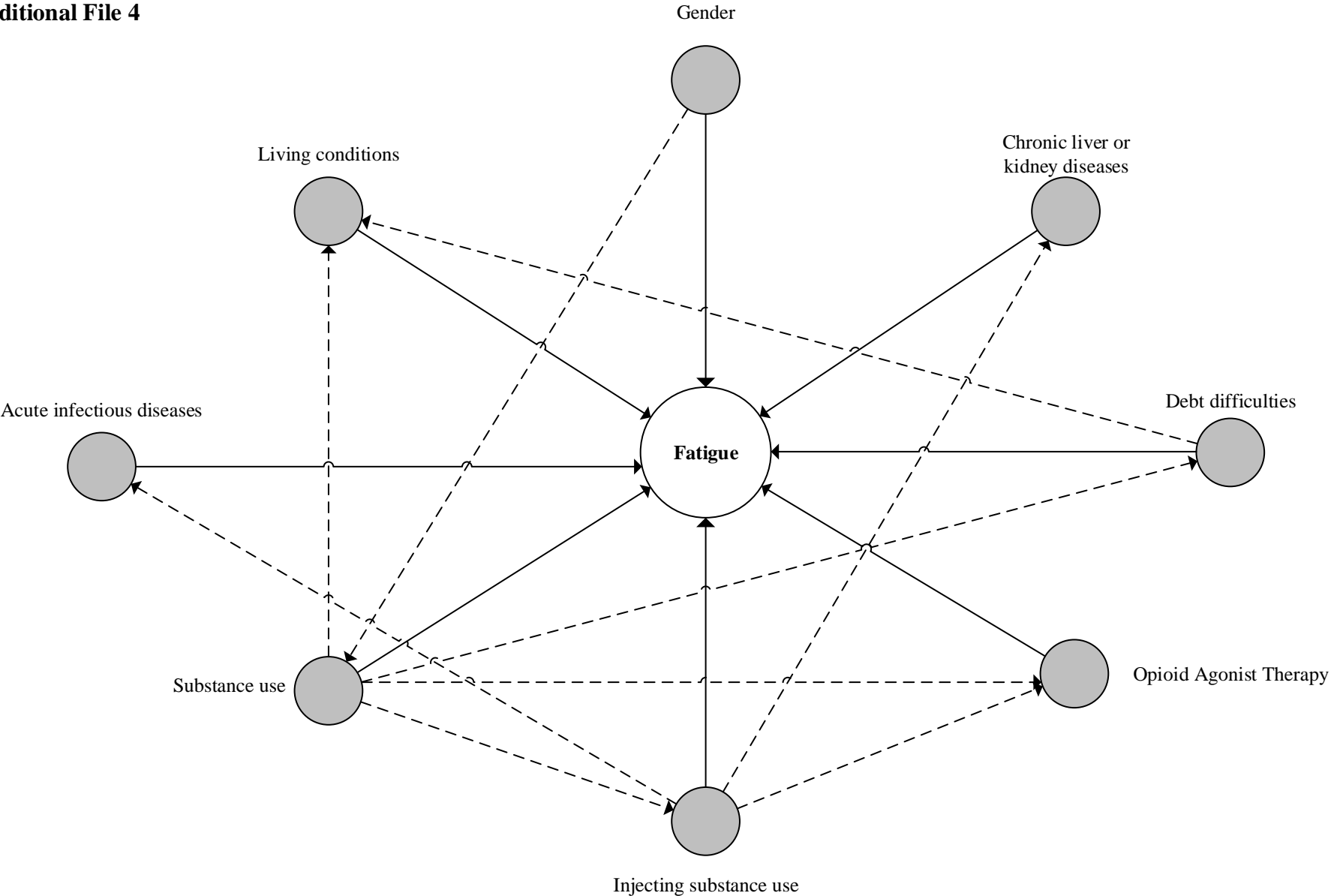

Supplement: Supplementary file 4 — Additional file 4. Potential correlations between sociodemographic and clinical factors and fatigue. The figure shows that potential sociodemographic and clinical comorbidities may affect fatigue among patients with substance use disorders. [file 13011_2020_334_MOESM4_ESM.pdf]
