# Supplementary material for: Impact of clinical and sociodemographic factors on fatigue among patients with substance use disorder: a cohort study from Norway for the period 2016–2020
Source: Subst Abuse Treat Prev Policy. 2020 Dec 14;15:93. doi: 10.1186/s13011-020-00334-x (PMC7737389; doi:10.1186/s13011-020-00334-x)
Supplement: Supplementary file 5 — Additional file 5. Mean (SD) item scores for single items on the FSS-9 at baseline and follow-up. Legend: FSS-9: Nine-item Fatigue Severity Scale; I: Item; OAT: Opioid agonist therapy; SD: Standard Deviation. Follow-up: The FSS-9 score on the last health assessment during the study period among patients with two or more annual health assessments. Mean (SD) item scores for single items on the FSS-9 for patients using methadone or buprenorphine as OAT opioid. [file 13011_2020_334_MOESM5_ESM.docx]

**Additional File 5**

|  | ***Methadone*** | | ***Buprenorphine*** | |
| --- | --- | --- | --- | --- |
|  | ***Baseline***  ***(N = 209)*** | ***Follow-up***  ***(N = 96)*** | ***Baseline***  ***(N = 321)*** | ***Follow-up***  ***(N = 107)*** |
| **FSS-9**  I1: My motivation is lower when I am fatigued | 5.5 (1.9) | 5.7 (2.0) | 5.4 (2.1) | 5.7 (1.9) |
| I2: Exercise brings on my fatigue | 5.0 (2.0) | 5.5 (1.7) | 4.6 (2.2) | 5.0 (2.1) |
| I3: I am easily fatigued | 5.0 (1.9) | 5.2 (1.9) | 4.5 (2.3) | 4.8 (2.2) |
| I4: Fatigue interferes with my physical functioning | 5.1 (1.9) | 5.5 (1.6) | 4.8 (2.2) | 5.0 (2.1) |
| I5: Fatigue causes frequent problems for me | 4.8 (2.1) | 4.8 (2.1) | 4.3 (2.3) | 4.5 (2.2) |
| I6: My fatigue prevents sustained physical functioning | 4.9 (2.0) | 4.7 (2.0) | 4.4 (2.3) | 4.1 (2.3) |
| I7: Fatigue interferes with carrying out certain duties and responsibilities | 5.3 (2.0) | 5.2 (2.0) | 5.0 (2.1) | 5.0 (2.1) |
| I8: Fatigue is among my three most disabling symptoms | 5.1 (2.2) | 5.1 (2.2) | 4.6 (2.4) | 4.8 (2.4) |
| I9: Fatigue interferes with my work, family, or social life | 5.1 (2.1) | 4.8 (2.2) | 4.9 (2.2) | 4.6 (2.2) |
| Mean score of all items | 4.8 (1.8) | 5.2 (1.6) | 4.7 (1.9) | 4.8 (1.7) |
| Sum score of all items | 43.2 (15.9) | 46.8 (14.2) | 42.5 (16.7) | 43.4 (15.6) |
