# Supplementary material for: Impact of clinical and sociodemographic factors on fatigue among patients with substance use disorder: a cohort study from Norway for the period 2016–2020
Source: Subst Abuse Treat Prev Policy. 2020 Dec 14;15:93. doi: 10.1186/s13011-020-00334-x (PMC7737389; doi:10.1186/s13011-020-00334-x)
Supplement: Supplementary file 6 — Additional file 6. Linear mixed model of fatigue (FSS-9) adjusted for sociodemographic and clinical factors among patients receiving methadone as an OAT opioid at baseline (N = 209). Legends: APRI: Aspartate transaminase to platelet ratio index; CI: Confidence interval; CRP: C-reactive protein; FSS-9: Nine-item Fatigue Severity Scale; eGFR: estimated glomerular filtration rate; HIV: Human Immunodeficiency virus; kPa: Kilopascal; OAT: Opioid Agonist Therapy. 1) Age per 10 years was centred according to mean age (43 years) in the study sample at baseline. 2) Includes amphetamine or cocaine use. The OAT opioid ratio: a ratio between the received dose of OAT opioids per day divided by the expected mean dose of OAT opioids (buprenorphine 18 mg, buprenorphine-naloxone 18/4.5 mg or methadone 90 mg). The educational level: highest level of education was coded 0–4 with 4 as the highest educational level. Unstable housing situation: living on the street, homeless shelter, or with family and friends at any time in the past 30 days prior to the health assessment. Debt difficulties: struggling with repaying current illegal and legal debt. Injecting substance use: Having injected a substance in the past 12 months prior to the health assessment. Frequent use of substances: at least weekly during the past 12 months prior to the health assessment. Viral load of HCV: From − 0.5 to 0.5, where the range ≥ − 0.5 to < 0 represents the low viral load (HCV PCR < 800,000 IE/ml), and the range from ≤0.5 to > 0 identifies the high viral load (HCV PCR ≥ 800,000 IE/ml). Zero (0) defined patients without HCV infection. When using the Bonferroni corrected p-values (αaltered = 0.05 / 43 = 0.0012), the predictors did not affect the FSS-9 sum score significantly. The table displays a linear mixed model analysis (Restricted Maximum Likelihood regression) evaluating sociodemographic and clinical factors’ (predictors) changes in the FSS-9 sum score at baseline and their influence on changes in the FSS- [file 13011_2020_334_MOESM6_ESM.docx]

**Additional File 6**

Linear mixed model of fatigue (FSS-9) adjusted for sociodemographic and clinical factors among patients receiving methadone as OAT opioid (N = 209)

|  | Fixed effects | | | | | | | |
| --- | --- | --- | --- | --- | --- | --- | --- | --- |
|  | Effect estimate | |  | | Time trend (per year) | | | |
|  | Estimate (95 % CI) | p-value | |  | Slope (95 % CI) | | p-value | |
| FSS-9 sum score | 48 (17 – 79) | 0.003 | |  | 29.0 (-8.6 – 66.5) | | | 0.129 |
| Female | *7.3 (2.5 – 12.2)* | *0.003* | |  | -2.8 (-8.9 – 3.2) | | | 0.358 |
| Age per 10 years^1)^ | -0.3 (-2.6 – 1.9) | 0.784 | |  | 1.1 (-1.9 – 4.0) | | | 0.486 |
| Educational level | -1.7 (-4.3 – 1.0) | 0.220 | |  | 1.7 (-1.6 – 5.1) | | | 0.304 |
| Unstable housing situation | 1.3 (-6.6 – 9.1) | 0.752 | |  | -1.1 (-11.6 – 9.5) | | | 0.844 |
| OAT opioid ratio | -0.4 (-7.7 – 6.9) | 0.909 | |  | -0.3 (-11.9 – 11.3) | | | 0.958 |
| Debt difficulties | *4.9 (0.7 – 9.1)* | *0.023* | |  | -0.4 (-5.9 – 5.1) | | | 0.885 |
| Injecting substance use | -0.2 (-5.1 – 4.6) | 0.922 | |  | -0.9 (-6.8 – 4.9) | | | 0.751 |
| *Frequent use of substances* | | | | | | | | |
| Benzodiazepines | *6.0 (1.6 – 10.5)* | *0.008* | |  | *-6.8 (-12.5 – -1.1)* | | | *0.020* |
| Alcohol | -0.8 (-6.0 – 4.3) | 0.747 | |  | 4.4 (-3.1 – 11.9) | | | 0.247 |
| Cannabis | 1.9 (-2.5 – 6.3) | 0.393 | |  | 3.0 (-2.7 – 8.6) | | | 0.300 |
| Opioids | 0.4 (-8.3 – 9.0) | 0.934 | |  | -1.2 (-13.9 – 11.6) | | | 0.858 |
| Stimulants^2)^ | -2.6 (-8.6 – 3.3) | 0.388 | |  | 2.8 (-4.6 – 10.2) | | | 0.451 |
| *Chronic infectious diseases* | | | | | | | | |
| Hepatitis B virus infection | 17.4 (-11.5 – 46.2) | 0.458 | |  | -10.6 (-26.2 – 4.9) | | | 0.179 |
| Hepatitis C virus infection   - Detected   - High. vs. low viral load | -12.5 (-33.8 – 8.8)  *31.5 (1.5 – 61.5)* | 0.249  *0.040* | |  | -  *-44.6 (-81.7 – -7.5)* | | | -  *0.019* |
| HIV | 10.7 (-8.4 – 29.8) | 0.272 | |  | 4.6 (-20.2 – 29.4) | | | 0.716 |
| *Liver stiffness* | | | | | | | | |
| Transient elastography per 10 kPa | 3.3 (-1.4 – 8.1) | 0.167 | |  | -*6.2 (-11.7 – -0.7)* | | | *0.027* |
| APRI score per 1 unit | -1.0 (-3.3 – 1.3) | 0.397 | |  | 1.6 (-1.0 – 4.1) | | | 0.235 |
| *Hematologic and biochemical blood samples (continuous variables)* | | | | | | | | |
| Hemoglobin per 1 unit (g/dL) | -0.5 (-1.9 – 0.9) | 0.458 | |  | | -0.5 (-2.3 – 1.3) | | 0.558 |
| eGFR per 30 units (ml/min/1.73m^2^) | -0.9 (-3.2 – 1.3) | 0.414 | |  | | 0.0 (-1.1 – 4.2) | | 0.463 |
| CRP per 10 units (ml/L) | -0.2 (-1.3 – 1.0) | 0.759 | |  | | 0.2 (-1.2 – 2.4) | | 0.834 |
